# Evaluation of an online arts-based platform to support the health and well-being of older adults during the COVID-19 pandemic: a cross-sectional survey

**DOI:** 10.1186/s12889-024-18720-6

**Published:** 2024-05-04

**Authors:** Justin Sutherland, Isabella Herrington, Julie Makarski, Jennifer Tindall, Mary Hynes, Monika Kastner

**Affiliations:** 1 Healthcare Management, Humber College, Toronto, ON Canada; 2 University of Toronto, Toronto, ON Canada; 3 Research and Innovation, North York General Hospital, Toronto, ON Canada; 4 Art Your Service Program, Toronto, ON Canada; 5 Institute of Medical Sciences, University of Toronto, Toronto, ON Canada; 6 Knowledge Translation and Implementation, Research and Innovation, North York General Hospital, Toronto, ON Canada; 7 Dalla Lana School of Public Health, Management, and Evaluation (IHPME), Institute of Health Policy, University of Toronto, Toronto, ON Canada

**Keywords:** Older adults, Arts-based intervention, COVID-19, Social isolation, Loneliness, Survey

## Abstract

**Objectives:**

The objective of this study was to conduct a formative evaluation of the *Art Your Service* (AYS) arts-based program to determine the program’s potential for improving the social and physical well-being of older adults during the COVID-19 pandemic.

**Design, settings and participants:**

An online questionnaire was administered to the AYS members who consented to be invited to participate in the study. Questionnaire items consisted of a Likert scale and open-ended questions delivered using an online platform (SurveyMonkey). Participants provided feedback on their perceptions and experiences of the AYS program, such as its impact on their health and well-being during the COVID-19 pandemic, the benefits and challenges of participating, and any suggestions for program improvement.

**Outcome measures and analysis:**

Quantitative data were analyzed using descriptive statistics (frequencies, means with standard deviations), and open-ended questions (qualitative data) were analyzed using content analysis. Outcomes included participant demographics, perceptions about the program, usability (System Usability Scale [SUS]), eHealth literacy (eHealth Literacy Scale), and social isolation (Lubben Social Network Scale; LSNS-6).

**Results:**

Program participants revealed consistent patterns of their perceptions and experiences about the program, including a high satisfaction rate (95%) and a perceived positive impact on participants’ health and well-being. The program sessions were perceived to be a well-organized, convenient, and safe way to engage with one another socially during the COVID-19 pandemic. The program usability was also perceived to be high (SUS mean score 86.2). Participants felt a sense of connectedness and had reduced feelings of social isolation. Most participants (75%) reported that the program improved their physical health.

**Conclusions:**

Findings from this formative evaluation study identified key strengths and opportunities to improve the *Art Your Service* arts-based program, which can be used to help enhance the program’s functioning and long-term sustainability potential.

**Supplementary Information:**

The online version contains supplementary material available at 10.1186/s12889-024-18720-6.

## Introduction

Arts-based interventions deliver activities to facilitate a creative experience for participants [1] in any arts-based discipline, such as performing arts (dance, music, theatre), community and cultural festivals, visual arts, design and craft, literature, creative writing, and online digital/electronic arts [2]. Arts-based interventions belong within the concept of creative arts therapies (CAT), which collectively refer to a wide range of arts disciplines such as art, drama, physical activity (through dance, movement, Yoga and Pilates), music and poetry aimed at promoting the creative processing of emotions and experiences [3, 4]. Arts-based interventions can be participatory (i.e., creating art such as painting, dancing, physical activity or playing an instrument) whereby individuals actively engage in the art or are directly involved in the art process. As such, arts-based interventions can also be considered as leisure-based because their participation is non-obligatory and recreational such through physical activity, gardening, or culinary arts [5]. Art-based interventions can also be receptive, whereby engagement in art is about observing or listening to an art form (e.g., watching a performance). Therefore, arts-based interventions can differ in their content and the type of interaction participants have. Whether participatory or receptive, art-based programs and interventions have been shown to promote healthy aging of older adults by improving their quality of life and well-being, cognitive functioning and memory; reducing cognitive decline [6, 7], frailty [8] and premature mortality [9]; decreasing depressive symptoms [10]; increasing their social interactions and connectedness [11–15] and reducing social disparities and injustices [16].

### Impact of social isolation and loneliness on the health and well-being of older adults

As adults age, their physical and cognitive capacity can progressively decrease, which can have a cascading effect leading to a breakdown or loss of social bonds, decreased social activities, and living alone. All of these factors can put older adults at increased risk of social isolation and loneliness [17] as well as decreased physical and mental well-being [15]. For example, social isolation and loneliness have been associated with coronary heart disease and stroke [18], type 2 diabetes [19], depression and psychological distress [20], dementia [21, 22], and impaired hearing [23]. Additionally, the mortality risk from social isolation is comparable to that of well-established risk factors such as smoking, lack of exercise, obesity and high blood pressure [24, 25]. Paradoxically, deteriorating physical and cognitive well-being also means that older adults need more social support to manage their daily lives or to cope with bereavement [26]. During the COVID-19 pandemic, older adults were identified as being at particular risk for social isolation during the public health mandates of physical distancing and quarantining to control the infection [27]. The risk of severe illness and mortality during the pandemic [28], as well as the need to maintain their mental health, highlighted the need to support the health and well-being of older adults in their homes [29].

### The potential of arts-based interventions to address the health and well-being older adults

There are promising arts-based interventions that can address the social isolation and loneliness experienced by older adults, but these are primarily delivered in-person or in groups. For example, the music-based Community of Voices Choir program, which was designed for ethnically diverse older adults with a range of singing abilities and experience, has been shown to reduce feelings of loneliness and increased interest in life [30]. In another example, an arts festival featuring older adult artists in music, literature, dance, theater performance and painting has shown to break down barriers and facilitate connectivity between those who were previously isolated such as in long-term residential care by promoting positive engagement with the community and other events held in these settings (i.e., day care centers, nursing homes and hospitals) [31]. However, there are few examples of arts-based interventions that can be considered during difficult situations requiring social isolation, such as the COVID-19 pandemic. For example, technology-based programs such as tele creative arts therapies (tele-CAT) have the potential to overcome geographical distances and accessibility barriers by reaching individuals with mobility disabilities that may prohibit their participation in live programs [4]. Keisari et al. developed a creative online intervention for community-dwelling older adults using photocollage [4] (a form of art therapy whereby participants select, arrange and attach various photographs to a blank surface), which enables participants to form new perspectives and to process memories and life experiences [4, 32]. However, to our knowledge, no arts-based intervention exists that offer both a diverse range of arts-based and other programs that can be widely accessible (via an online platform) by older adults with variable physical, cognitive and social functioning. A recent systemic review investigated arts-based technology interventions but it targeted older adults with cognitive impairment and dementia only [33]. Additionally, most existing arts-based interventions involve a single type only (e.g., visual arts, music, dance, fitness), lacking the diversity of activities that would cater to the broad interests of older adults [33]. Our study fills this gap by investigating the impact of an innovative arts-based, online program called “Art Your Service” (AYS) that was created for older adults during the COVID-19 pandemic. The objective of our study was to conduct a formative evaluation to determine the perceptions of AYS members about the program’s potential to improve their social, psychological, and physical well-being during the pandemic.

## Methods

### Intervention

The AYS program was developed in Whitby Ontario, Canada by a group with a background in special events, arts programming, and aging. It started as an in-person program in 2019 aimed at building a community for older adults to engage in arts-based services and events in Canada [34]. The program started off in person to provide physical programs to retirement homes and social events at art galleries, but it did not have a regular in-person schedule. Realizing the immediate need for older adults to be engaged in arts-based activities in response to the COVID-19 pandemic, the AYS program was re-designed as an online, virtual platform (August 2020) to offer physical activity and creative sessions. Its aim was to address the loneliness and isolation that many older adults experienced due to mandated public health restrictions. Since August 2020, the AYS program offers various live and archived (video) virtual classes (via Zoom) including a range of arts-based activities, from dance, yoga, meditation, Pilates, art and music to creative aging and virtual museum visits. The average number of attendees per session is about 75 older adults. The classes offer a wide range of content, from fitness and movement to creative classes, including singing and painting. In 2022, the virtual platform also launched the “Caregivers’ Corner” (a partnership with Niagara, George Brown and Sheridan Colleges in Ontario), which provides movement, cognitive and creative videos (developed by college students) to support caregivers and staff in long-term care facilities.

### Study design, population and recruitment

To address our objective to determine older adults’ perceptions of the AYS program’s potential impact during the COVID-19 pandemic, we conducted a closed, online, cross-sectional questionnaire (SurveyMonkey) using the Checklist for Reporting Results of Internet E-Surveys (CHERRIES) evaluation framework [35] with older adults who were AYS program members. The questionnaire was administered from November 4 to December 19, 2021. We used a purposive sampling strategy to recruit older adults (age 50 +) who were members of the AYS program at the time of our study (~ 400 older adults). Due to research and ethics board (REB) constraints in directly inviting the ~ 400 members of the AYS program, we had to first seek members' approval to be invited to participate in the study. We received consent from 97 individuals to be invited to participate in the questionnaire study, which formed our sampling frame. REB approval was obtained through the North York General Hospital in Toronto, Ontario, Canada. The study participation informed consent process was embedded within the online questionnaire, whereby the introduction page asked potential participants to review the study information and accept or decline consent to participate. Once they did, the questionnaire navigated them to the first question. Those who declined to participate were asked to provide their reason. We used Dillman et al.’s follow-up strategy to maximize our response rates (i.e., three reminders, two weeks apart, followed by a personalized email to non-responding mentor and mentee participants) [36]. To maximize recruitment, we offered gift cards to be won during the six weeks the questionnaire was open (November to December 2021).

### Questionnaire development

The online, closed questionnaire was developed in collaboration with the study team to elicit an understanding of participant characteristics; and to assess the impact of the AYS program on participants’ social, psychological, and physical well-being during the COVID-19 pandemic. The questionnaire included a combination of Likert-type and open-ended questions. The following sections were included in the questionnaire (one section per page): (i) participant demographics (age, sex, race, employment status, relationship status, education, income, postal code); (ii) social connections (Lubben Social Network Scale [LSNS-6] [37]); (iii) general health (overall health, mental or emotional health); chronic conditions; eHealth literacy [38]; (iv) AYS program access and use (how often, in what format and when program is used, how long the program has been used, which classes accessed and participated in, future topics, reason(s) for joining the program, whether the program has met their needs; (v) perceived impact of the AYS program on their health and well-being; (vi) program delivery (difficulties with access; challenges, advantages and benefits to participating, session delivery; and (vii) usability of the AYS program (System Usability Scale [39]); and (viii) satisfaction with the AYS program. The questionnaire was pilot tested with three older adults (not involved in the questionnaire study) to ensure appropriate flow and clarity of questions, questionnaire completion time, and any suggestions for improvement prior to launching the questionnaire. All participant data were coded using a unique identification number that was automatically generated by our online questionnaire platform (Qualtrics). Any duplicate database entries were eliminated prior to analysis by comparing user identification numbers. Some items used adaptive questioning (e.g., conditional items) to reduce the number and complexity of questions. Given the number of questionnaire sections and questions, we did not randomize items. Response options included ‘Not applicable’ and most items were forced to maximize questionnaire completion. All results were presented in aggregate form to keep individual responses confidential.

### Analysis

Data from fully or partially completed questionnaires was analyzed using descriptive statistics. This involved calculating frequencies, as well as to assess central tendency (means, median and standard deviations) and level of dispersion using interquartile range (IQR), which provides an assessment of the extent of agreement between participants (i.e., IQR of 0, 1 and 2 are considered high, good and poor consensus; respectively). We performed a chi-square test of independence to investigate any associations between variables using Jamovi 1.6 statistical software. We also plotted means and their SDs using bar graphs for quantitative data (i.e., Likert-type questions). Open-ended questions (qualitative data of respondent perceptions) were analyzed using content analysis [40]. Qualitative data were used to support our interpretation of the quantitative data.

### Outcomes and measures

Quantitative outcomes included a measure of social isolation using the 6-item Lubben social network scale (LSNS-6), whereby higher scores (ranging from 0–30) indicate more social engagement [37]. We used Lubben et al.’s cut point of LSNS-6 subscale score < 6 to indicate isolation from family or friends and a full-scale score of < 12 to indicate risk for social isolation [37]. General health was assessed using questions from the EQ5D, including a visual analogue scale [41]. eHealth literacy was assessed using the 8-item eHEALS scale [42] The overall scores can range between 8–40, with higher scores indicating greater perceived skills at finding, evaluating, and applying eHealth information to make health decisions [42]. We used the median eHEALS score as the cut point for low or high eHealth literacy (i.e., scores above the median were considered high eHealth literacy and scores below the median were considered low) [43]. Perceived overall health and mental or emotional health were assessed using a 5-point Likert scale (where 1 = poor, 2 = fair, 3 = good, 4 = very good, and 5 = excellent). We assessed the usability of the AYS program using the System Usability Scale (SUS), which can be used to evaluate websites and applications [39]. A SUS score > 68 is considered above average, > 70 is considered acceptable, an adjective score of > 74 is considered excellent, and > 85 is considered best imaginable [44]. We used Bangor et al.’s adjective rating scale to enhance a reviewer’s ability to better translate the SUS score (0–100) into an absolute judgement of usability thereby providing a clearer picture of the innovation being tested [44].

## Results

### Participant characteristics

Of 97 individuals who agreed to be invited to the study, 71 older adults consented to participate (73% response rate). Questionnaire completion rate across questions varied from 87–100%. The mean age of participants was 72 years (range 49–91 years) (Table 1). The number of female participants outnumbered male participants (91.5% vs. 8.5%, respectively). The majority of older adults were from Ontario, Canada (96%), identified as white (89%), retired (82%), and having an undergraduate or college degree (63%). Almost half of respondents were married (46.5%) and 38% were living alone. Respondents perceived their overall health and their mental or emotional health as good (mean scores of 3.5 and 3.4, respectively). Participants reported having high blood pressure (38%), osteoarthritis (34%), depression (21%), high cholesterol (20%), diabetes (11%) and osteoporosis (11%); 14 individuals (20%) reported having no health conditions. The mean LSNS-6 social network score was 16. We identified 22 respondents (31%) who had limited family-based social networks (LSNS-6 score < 6) and 26 respondents (37%) who had limited friends-based social networks. There were 17 respondents (24%) who were identified as being at risk for social isolation LSNS-6 score < 12). The mean and median eHEALS scores among the 71 respondents were 29.7 and 31, respectively, which meant that 39 respondents (55%) were considered to have high eHealth literacy.

**Table 1:** Participant characteristics of survey respondents

Characteristic	N (%)
**Age (*n* = 69)**	
Mean age	72 years
Age range	
49–54	5 (7.2)
55–64	7 (10.1)
65–74	32 (46.4)
75–84	22 (31.9)
85–94	3 (4.3)
**Sex (*n* = 71)**	
Female	65 (91.5)
Male	6 (8.5)
**Province/State (*n* = 71)**	
Ontario, Canada	68 (95.8)
Alberta, Canada	1 (1.4)
Nova Scotia, Canada	1 (1.4)
Arizona, USA	1 (1.4)
**Race (*n* = 71)**	
White	63 (88.7)
Black	5 (7.0)
Arab	1 (1.4)
Indigenous	1 (1.4)
Other: Multiracial	1 (1.4)
**Disease**^a^	
High Blood Pressure	27 (18.75)
Osteoarthritis	24 (16.67)
Depression	15 (10.42)
High Cholesterol	14 (9.72)
None	14 (9.72)
Other^b^	13 (9.03)
Diabetes	9 (6.25)
Osteoporosis	8 (5.56)
Urinary Incontinence	6 (4.17)
Heart Disease	5 (3.47)
Lung Diseases	3 (2.08)
Heart Failure	2 (1.39)
Rheumatoid Arthritis	2 (1.39)
Chronic Kidney Disease	1 (0.69)
Stroke	1 (0.69)

^a^Participants could indicate multiple responses

^b^Other diseases were: muscle damage and weakness, asthma, glaucoma, chronic dizziness, migraines, Sjogren’s disease, spinal fusions, breast cancer, eye problems, acquired brain injury, Vogt-Koyanagi-Harada Disease, cancer, prostate cancer, multiple sclerosis

### AYS program evaluation

Results of the formative evaluation of the AYS program was organized according to three major domains: 1) Characteristics of the AYS program; 2) Perceived impact of the AYS program; and 3) Satisfaction with the AYS program.

#### Characteristics of the AYS program

##### AYS program delivery

**Fig. 1:**
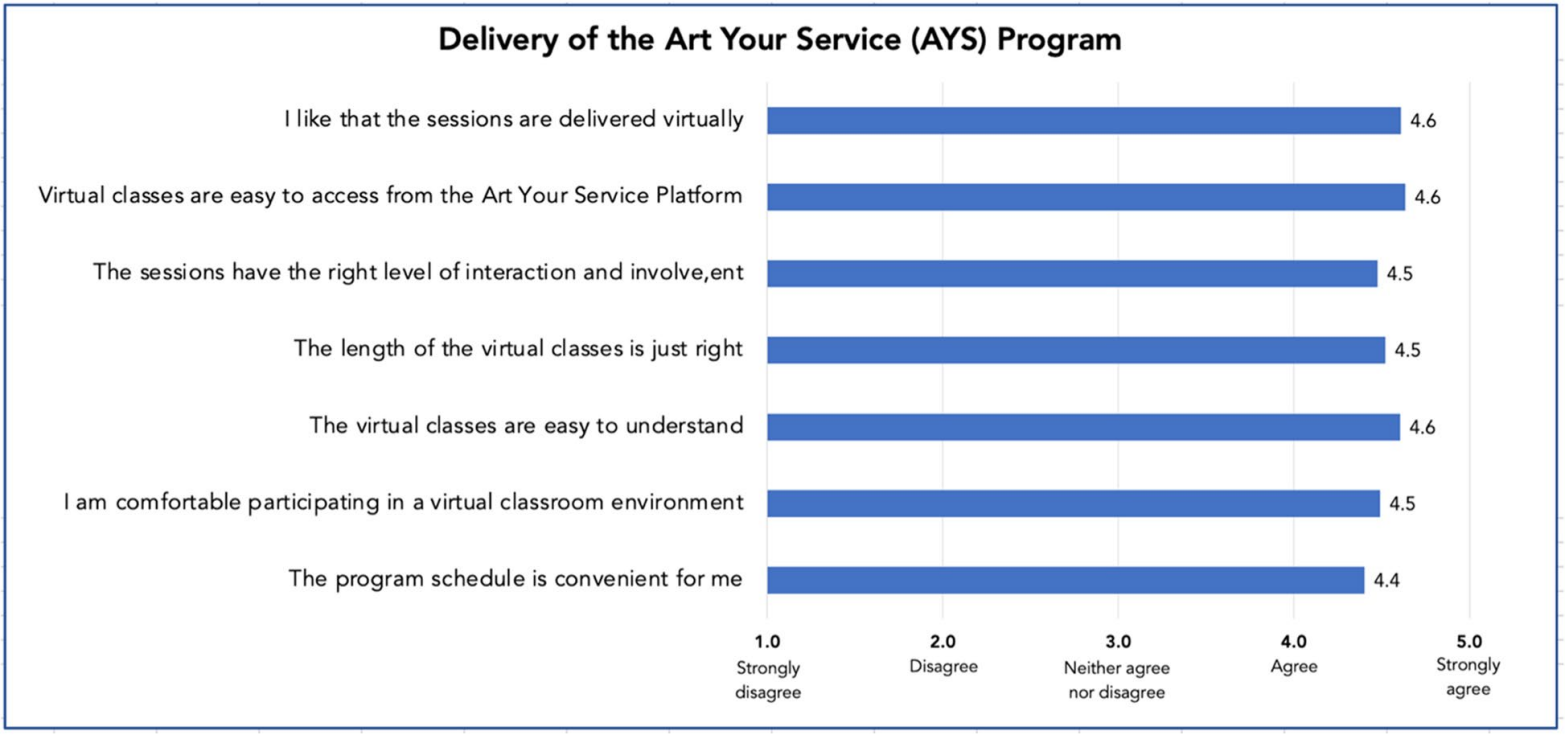
Characteristics of the AYS program delivery

Questionnaire respondents became aware of the AYS program mostly through a Seniors Centre or their Municipality (45%) or were referred by friends or family (32%) (Appendix A). Nearly half of respondents (48.5%) used the AYS program for 12 months or more at the time of the questionnaire. Most respondents used the program three or more times per week (57%), primarily in the mornings (46%) and afternoons (26%). Most respondents (56%) engaged in both live and recorded (archived) classes (Appendix A). Respondents liked that the classes were delivered virtually and found this format comfortable and easily accessible from the AYS platform (mean score range 4.5–4.6 out of 5). Sessions were perceived to be easy to understand, with the right length and level of interaction and involvement, and the program schedule was convenient (mean score range 4.4–4.6) (Fig. 1). Most respondents joined the AYS program to improve their physical fitness (81%), to learn something new (76%), to help get through difficult situations such as the COVID-19 pandemic (61%), and to improve their social and emotional (52%) and mental (52%) well-being (Appendix A). Respondents also joined the AYS program to interact with others (45%) or because they felt isolated (42%), bored (39%), or lonely (27%). Most respondents (87%) indicated that their goals for joining the AYS program had been satisfied (Appendix A). The central tendency and spread of Likert-scale scores showed good agreement between participants for all items related to AYS program delivery (IQR 1) (Appendix B).

##### AYS program content

The most frequently accessed classes were physical activity related: *Gentle Moves Dance* (62%), *Joy of Movement* (62%), *Pilates* (55%), *Seated and Standing Fitness* (54%), and *Yoga and Meditation* (52%) (Appendix C). Additional topic areas that were suggested by respondents to be included in the AYS program were: *board & card games, financial literacy, peer connections, educational, arts-based, mindfulness, physical activity, nutrition, and hobbies/interests. Mind-stimulating games such as Trivia, Chess, and Yahtzee were suggested as additional activities*. Some participants were interested in improving their financial literacy, suggesting courses on taxes or banking tips for seniors. Other educational recommendations included lectures from doctors and astronauts, technology use for seniors, and Métis culture and language courses. Participants expressed interest in learning new arts-based activities such as playing specific instruments, drawing, and learning how to do watercolour. Some respondents suggested mindfulness exercises to prevent boredom, such as longer meditation sessions. Physical health-related classes, such as *Tai Chi, Seniors Zumba, and Osteofit,* were also encouraged. Some participants suggested classes based on their personal hobbies and interests, such as ancestry photography, travel discussions and animal-related activities. The class instructors were generally perceived as excellent (Appendix C):

* “I think the instructors do an excellent job of ensuring the content is relevant to all participants ... i.e., using a chair versus standing. And more aggressive versus less aggressive. Well done!”*

##### Usability of the AYS program

Overall, participants perceived the AYS program to be user-friendly. The total mean SUS score was 86.2 (*n* = 65, standard deviation = 11.1), which showed that the overall usability of the AYS program was perceived as “best imaginable” [44]. Nearly all participants (92%) indicated that the biggest advantage of the AYS program was that it does not require travel: *“It [AYS program] has given me the opportunity to join many different types of programs without leaving my home.”* Other benefits shared by respondents were improvement in their physical health (82%), flexible timing of the classes (79%), ability to practice at their own pace (74%), ease of access (72%), and perceived improvement in their cognitive health (thinking skills) (63%) as well as their mental and psychological health (loneliness, isolation connectedness) (52%) (Appendix D).

##### Challenges and barriers to the AYS program

Most participants (83%) did not report any challenges to participating in the AYS program, and 62% did not have any difficulties using it (Appendix E). There was a significant association between eHEALS score and experiencing barriers or challenges to participating (*X*^*2*^ (1, *N* = 71) = 5.62 *p* = 0.018). Specifically, participants with low eHealth literacy were more likely to report encountering barriers or challenges compared to those with high eHealth literacy (Phi-coefficient = 0.281, Cramer’s V = 0.281), indicating a moderate association. Participants that did have difficulties using the AYS program, were related to Internet connectivity/network problems (27%) and sound quality (17%), attributed mostly to *“occasional network and internet challenges”*.

Some participants described that poor computer skills or fear of using the computer for new things may limit their use of the AYS program and not be able to access its full potential:

*“I have enjoyed most of the programs I have seen, but I do not take an active part because of poor computer skills.”*

*“I think that “fear of trying new things on a computer” is more of a barrier for seniors. I joined because a friend invited me. I have invited my friends, but only one joined for a short while.”*

##### Suggestions for improving the AYS program

Overall, participants offered suggestions for improvement to the AYS program, ranging from additional tech features and usability support to arts-based activity clarity and instructor delivery. Suggestions were made to improve some of the accessibility features, such as increasing the font size of the chat feature to help the readability of messages for users who are visually impaired and increasing the size of the video when accessing recorded classes. Other suggestions included being able to click on a class that would automatically add it to their daily calendar and providing technology education and assistance as well as one-on-one workshops for those with limited technical skills or those who experience barriers to technology use. In terms of course content, some participants would have appreciated more meaning and clarity behind paintings during painting sessions, while another participant had expressed a preference for more “instructor movement rather than verbal instruction.” Participants also expressed their desire for more convenient time options for the classes and to offer classes on the weekends and during the evening: *"I would like to participate in some of the more social programs, but my caregiving duties do not allow for many real-time sessions."* To facilitate more interaction among the AYS program users, one recommendation was to make a chat group available for AYS program members so they could converse with each other.

#### Perceived impact of the AYS program

##### Perceived impact of the AYS program on health, well-being and quality of life

**Fig. 2:**
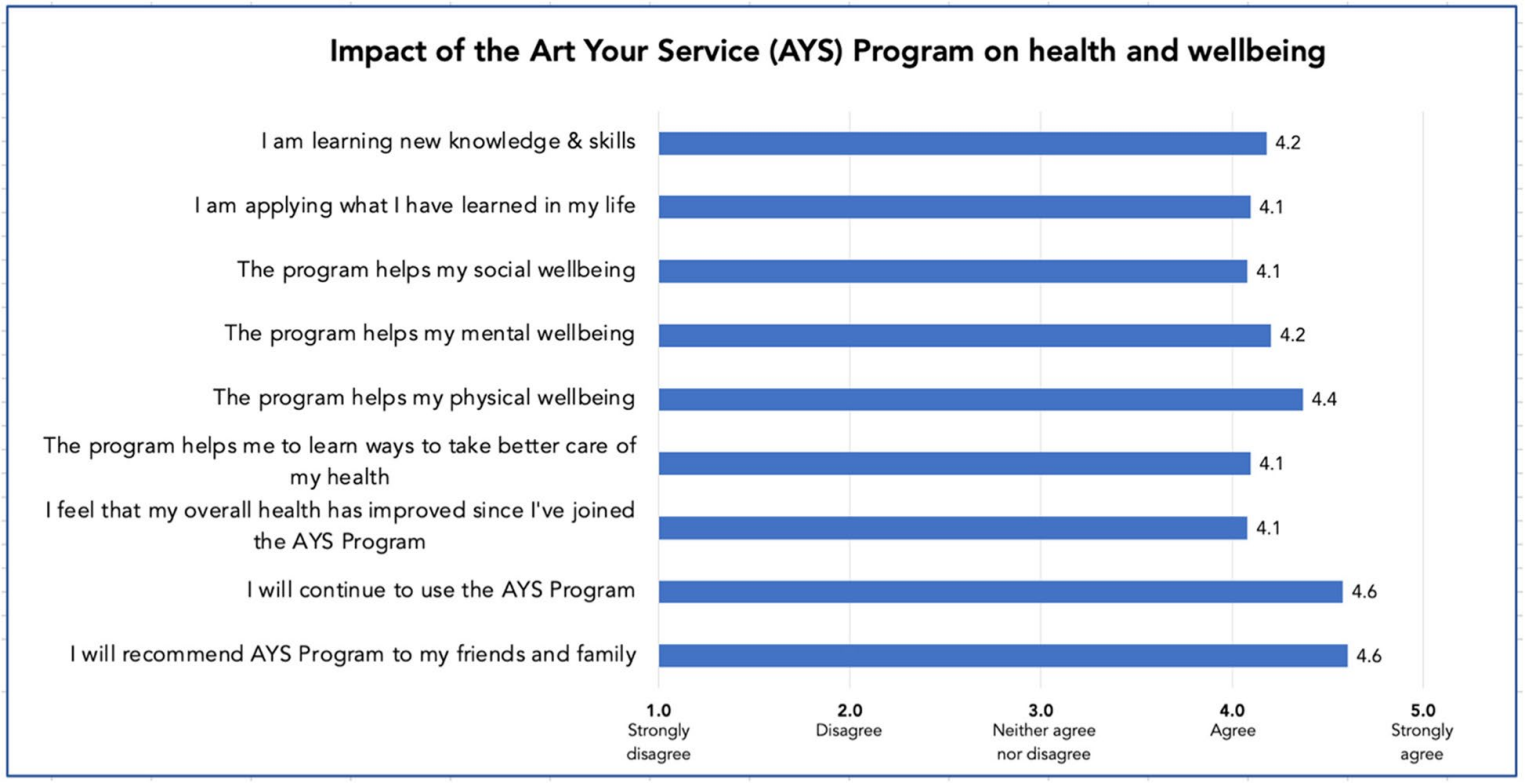
Perceived impact of the AYS program on health and well-being

Table 2 shows the central tendency and spread of Likert-scale scores showing good agreement between participants for all but one item pertaining to impact on health and well-being *(‘I feel that my overall health has improved since I’ve joined the AYS Program’*). Respondents agreed that the AYS program helped their social, mental, and physical well-being and improved their overall health (mean score range 4.1–4.4 out of 5) (Appendix B). They also learned new knowledge and skills and were applying what they have learned through the program in their own life (mean score range 4.1–4.2 out of 5) (Fig. 2). The program also informed participants of what they needed to do to improve their health, well-being, and quality of life. Participants felt that the AYS program made them more physically active and physically stronger and improved their mobility and balance (Appendix F):

* “[The AYS Program] had made a great deal of change on my physical well-being that allowed me to gain the physical strength required to continue to live on my own.”*

*“My mobility has increased immensely; I can care for myself so much better, i.e., in tying my boots/shoes; reaching; not falling or being able to stop myself from a fall due to increased balance.*”

##### Perceived impact of the AYS program on social and emotional wellbeing

Overall, the AYS program motivated participants to engage socially and to participate in more activities to improve their social, emotional, and mental health. In addition, respondents indicated that the Zoom sessions helped them to reduce their loneliness and lessened their isolation:

* “[AYS] has kept me active and brightened my spirits when I have the lost feeling after the loss of my husband after 55 years.”*

*“The Zoom sessions helped me work through the recent loss of my husband by meeting new people.”*

*“They [AYS] have made a great deal of change to my physical and therefore my mental well-being they have allowed to get the physical strength I require to continue to live on my own.”*

*“Participating in the Zoom sessions helped overcome the slight loneliness due to the lack of group activities in my retirement community. Physically, the sessions were a godsend, as I am motivated by being part of a group.”*

##### Perceived impact of the AYS program during the COVID-19 pandemic

Overall, the AYS program provided participants with a convenient and safe method to engage socially while managing their physical and emotional health during the COVID-19 pandemic lockdowns. Nine major themes were generated from the data (Appendix G):

1. *A sense of routine, structure, and purpose during lockdowns*: The AYS program provided participants with a sense of routine and structure. The classes kept them busy with scheduled classes and gave them something to look forward to:

*“It has been wonderful to get up in the morning and attend an easy-going exercise, then maybe at 2:00 pm take a relaxing painting class. It certainly filled in a gap; instead of watching TV all day and also learning new skills and hobbies, I will continue.”*

*“The structure of having an exercise class each morning helps me get my day going. The library helps me be productive and relaxed when I need that.”*

*“The sessions were a godsend during the lockdowns. They provided a routine during the dreary winter months and motivated me to do physical exercise.”*

(2) *Accessibility and convenience*: Respondents found the AYS program convenient since participation does not require travel and allows for self-directed access. In addition, they had access to the online program library if they missed a session:

* “I personally feel that this is a great way to participate in classes as you set your own schedule and have access to the library when live class is missed.”*

Participants also appreciated the variety of activities offered that did not require leaving their homes and facilitated their social interaction, despite geographic barriers.

(3) *Distraction from the pandemic*: The AYS program served as a positive diversion and distraction from ongoing and distressing news about the COVID-19 pandemic:

* “The daily programs provided something to do, a distraction from the ongoing news cycle [about COVID-19].”*

*“Kept me focused from not always having Covid on my mind”*

“*I am appreciative that I am able to have a group to go to and not have to worry about covid or lockdowns. It provides me with a healthy distraction.”*

(4) Something to look forward to: Many participants described the AYS program as providing something to look forward to, particularly during lockdowns:

* “Something to look forward to during the afternoons, as/an alternative to knitting in front of the TV.”*

*I just really look forward to participating and learning. Otherwise, I would just be watching TV and talking to the dog, LOL.”*

(5) *Emotional stability and support:* Participants indicated that the AYS program provided emotional stability and support that got them through the COVID-19 pandemic and described it as a *“bright spot during a dark time.”* Others described the program as making them feel happier and better and to be able to respond more positively to the pandemic:

* “It helps me get through covid as well as it helps me temporarily escape from the senior’s residence where I live…”*

*“I have remained relatively sane during this pandemic, and I do believe that coming to this site has made a big difference for me.”*

(6) *Filling a void*: Participants felt that the activities available through the AYS program filled an important void in their daily routines:

* “It has been wonderful, just to get up in the morning and attend an easy-going exercise, then maybe at 2.00pm take a relaxing painting class. It certainly filled in a gap, instead of watching tv all day…”*

*“Something to do since all my volunteer and exercise activities were cancelled.”*

(7) *Learning something new*: Some participants experienced a boost in confidence as they learned new skills throughout the program activity offerings. *“I have learned new and interesting skills that make me feel confident to try new things.”.* Additionally, many respondents perceived the value of the AYS program as providing the opportunity to “learn something new” and to develop new interests during lockdowns:

* “This program has brought a lot of joy to my life...and the joy of movement and the stimulation of ongoing learning...I am learning to play the ukelele and I want to learn to paint. Priceless.”*

*“Art Your Service has been a great way to continue to exercise and to learn new programs that I would have missed during the lockdown. They are so good that I really enjoy the variety of classes offered.”*

*“At first AYS was a real substitute for my loss of participation in and outside the home activities -- I missed all the fun things I had been attending at the Seniors Centre and at church. I could join by zoom to such a variety of programs every day. Something to look forward to. Then I connected with others attending the classes, got to know them a bit, looked forward to seeing them. Shared a few laughs, etc.”*

*“I just really look forward to participating and learning. Otherwise, I would just be watching TV and talking to the dog, LOL.”*

(8) Motivation to maintain health: Participants also indicated that the AYS program provided a source of motivation to spend time engaging in program activities and to maintain their overall health and well-being:

* “The sessions were a godsend during the lockdowns. They provided a routine during the dreary winter months and motivated me to do physical exercise.”*

*“It has helped me mentally, physically and emotionally respond to the covid pandemic in a positive manner and enriched my life beyond measure.”*

(9) Opportunities for social interaction and connectedness: The AYS program was perceived as beneficial in helping participants connect with others. They appreciated the social interaction and connectedness opportunities that the AYS program provided, which reduced their feelings of social isolation:

* “The boredom and sadness of the lockdown was so diminished the more classes I took. I felt busy and inspired, and purpose was back in my life.”*

*“I have met a variety of interesting and talent people and feel connected to the outside world...I have empathy for others in different situations…”*

*“I am grateful for the program. As a living alone senior, in seniors building, COVID-19 caused instant isolation.”*

*“Before Covid I would have at least 6 lunches out amongst others, go on bus trips & cruises so AYS fills a gap in my social life.”*

#### Satisfaction with the AYS program

**Fig. 3:**
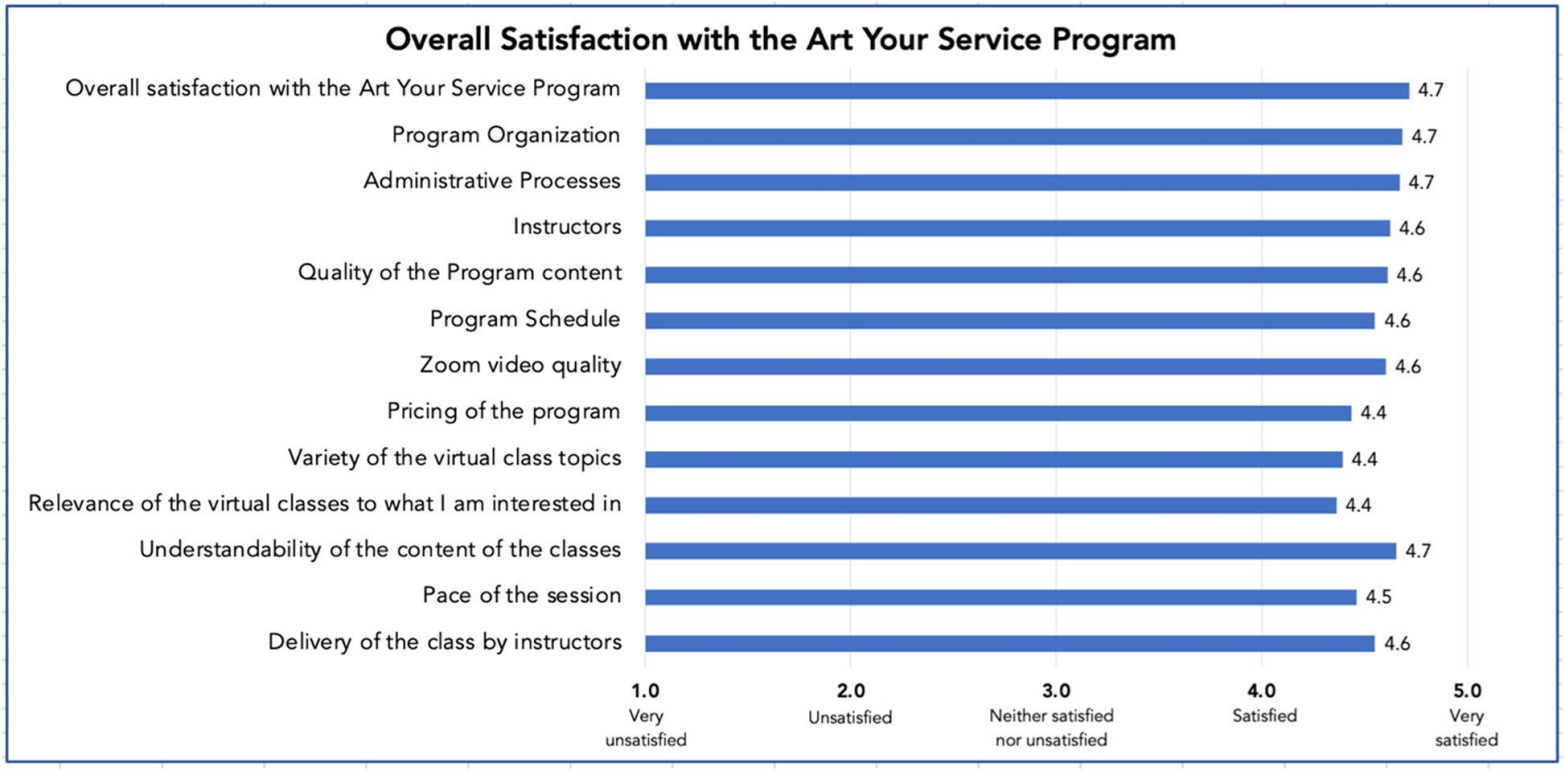
Overall satisfaction with the AYS program

Appendix B shows the central tendency and spread of Likert-scale scores showing high to good agreement between participants for all items pertaining to AYS program satisfaction (IQR range 0–1). Respondents were very satisfied with the AYS program overall (mean score 4.7 out of 5). More specifically, respondents were satisfied with the program organization, administrative processes, session instructors and delivery, the pace of the sessions, the quality of the program content, Zoom video quality and the program schedule (mean score range 4.5–4.7 out of 5) (Fig. 3). One participant expressed their satisfaction with their interactions with the program director and instructors: “*It is excellent! They have the best staff and instructors! Kind, caring and very helpful. Everything is paced to the senior.”* Participants were also satisfied with the variety of class topics and expressed appreciation for the diversity of topics. Additionally, they appreciated the relevance of the topics to what they are interested in and the understandability of the content (mean score range 4.4–4.7 out of 5) (Fig. 3). They found value in the program's many components and recommended it to others: *“I have spent so much time on this Program. I am so glad the Program was offered to me by my Municipality. I cannot express the huge difference the Program has made in my life. The Program is well organized, and every member is treated with respect and kindness.”* Additionally, many respondents indicated a strong desire for the AYS Program to continue into the future: *“I hope Art Your Service Program will continue. I cannot imagine my life without it.”*

## Discussion

We evaluated the AYS program to determine the perceptions of its members on the potential impact of the program on their social, psychological, and physical well-being, particularly during the COVID-19 pandemic. Our questionnaire analysis showed consistent patterns of perceptions and experiences of the AYS program: questionnaire respondents were very satisfied with the program, and most were motivated to engage in social and physical activities, which had a perceived positive impact on their physical, social, and emotional health and well-being. Participants found value in the program's many components, and virtual sessions were perceived to be well-organized and convenient. The overall program was perceived by participants as a safe, convenient method to take part in activities while managing their physical and emotional health during the COVID-19 pandemic lockdowns. The overall usability of the program was rated high (SUS mean score 86.2), which was consistent with participants’ perceptions of their experience, satisfaction and improved health and well-being from using the program overall. Most questionnaire respondents also expressed appreciation for feeling a sense of connectedness and reducing their feelings of loneliness and social isolation because of using the AYS program.

### Strengths and limitations

Our study had several strengths. First, we evaluated the potential impact of an innovative, online arts-based program targeting older adults, which is needed to address and alleviate the approximately 30–50% of older adults who experience social isolation and one-third that experience some degree of loneliness [24, 45–47]. Older adults who live alone have an increased risk of social isolation and loneliness [17] as well as decreased physical and mental health well-being [15].

In Canada, solo dwellers represent 42% of all people aged 85 years and older [48]. This is consistent with our results, which showed that 38% of our study sample reported living alone, 31–37% had limited family or friends-based social networks, and 24% were identified as being at risk for social isolation (an objective measure of the quantity and sometimes the quality of social relationships [18, 49] such as having few contacts or living alone). We need innovative solutions such as the AYS program to prevent or reduce the negative effects of social isolation, particularly during difficult situations requiring social distancing and isolation such as the COVID-19 pandemic. Our evaluation study contributed to filling this gap by providing evidence of the potential benefits of an online arts-based intervention on the wellbeing of older adults. The AYS program is a unique, online solution that engages older adults in a wide range of arts- and physical activity-based classes offering live and archived content. Most existing arts-based programs are focused on a single activity (e.g., fine arts, dance, music, fitness) or delivered in-person, which makes it less accessible to older adults who may not have the capacity to attend in-person classes or live in rural areas.

Second, our findings showed that the AYS program not only benefited older adults to engage socially during the COVID-19 pandemic, but it was also a source of attaining physical health. Our findings showed that older adults are interested in and want to engage in physical activities and like the flexibility and variety of classes that an online platform such as AYS can offer. In fact, the most frequently attended classes that older adults accessed were physical activity related such as dance, Pilates, seated and standing fitness and Yoga. This level of interest was also aligned with respondents’ reason for joining the AYS program, which was to improve their physical fitness (81%). This is not surprising as the benefits of physical activity on the health of older adults are well established. It contributes to independent living, and leads to improvements in physical and mental health, cognitive functioning, quality of life and emotional, psychological, and social well-being [50–52].

Third, the results of this study can also benefit self-management program developers by highlighting the specific factors of arts-based programs that may appeal to older adults. By understanding these factors, program components can be carefully constructed to alleviate the social isolation and loneliness often experienced by this population group. As demonstrated by the AYS program, offering a wide range of art forms (i.e., music, dance, literary arts, visual arts, and craft events) can appeal to a wider range of participants with different activity preferences. Third, our study contributes to the investigation of the potential for arts-based interventions to improve the mental and physical well-being of older adults. Many respondents indicated that the AYS program aided their self-management, which presents an opportunity to integrate AYS with other e-health and self -management technologies or alongside clinical management strategies to support overall health and well-being. Lastly, results from this study have shown high satisfaction among program participants during and after the COVID-19 Pandemic, indicating that it would be of benefit to older adults regardless of public health circumstances.

Our study also had some limitations. First, we were not able to recruit all members of the AYS program (~ 400 members at the time we conducted the study), so our sample may not represent all older adults who have used the program, and therefore limit the generalizability of findings. Although our questionnaire participants were a bit younger compared with the AYS members (mean age 72 vs 76 years of age, respectively), the sex distribution of mostly female users of the program (92% vs 95%, respectively) and residing mostly in Ontario (96% vs. 93%, respectively) were comparable. Additionally, we had a high response rate (73%) among the 97 members who consented to complete the questionnaire. Second, the AYS program is currently only available in English and for individuals who have technology (computers, tablets, smartphones) and internet access, which may limit its accessibility. However, future updates to the program will involve addressing these barriers. Third, we cannot yet make definitive conclusions about the effectiveness of the AYS program without conducting a more rigorous evaluation study, such as with a randomized controlled trial. In a scoping review investigating creativity and art therapies to promote healthy aging in older adults found that of the 12 included studies that used a quantitative design (*n* = 7), most were a pre-/post, questionnaire or longitudinal design, and the remainder were qualitative (*n* = 4) or mixed-methods (*n* = 1) designs [12]. Overall, our findings are consistent with existing art-based programs in terms of their potential to promote healthy aging by improving participants’ quality of life and well-being, and increasing their social interactions and connectedness [9, 11–15]. As a first step, our increased understanding of the program activities and content from this study showed potential to improve the well-being of older adults, which will allow us to improve and enhance the AYS program overall (e.g., translation of the program into other languages and increasing access).

## Conclusion

Findings from this evaluation identified key strengths and opportunities to improve the Art Your Service (AYS) program participant outcomes, participant perceptions, program usability, and program delivery. Perceived strengths of the program were its virtual delivery, usability, and the positive impact on participants’ feelings of loneliness and social isolation as well as their perceived improvement in their physical, social, and emotional well-being. Suggestions for improvement were to add more technological and accessibility features, include more usability support, more convenient time options and more opportunities for interaction among AYS members. Results will be useful to (i) understand what factors, activities and content can best alleviate the social isolation and loneliness experienced by older adults during and after the COVID-19 pandemic; and (ii) use findings to optimize the *Art Your Service* (AYS) program. Further research will be conducted to explore the potential effectiveness of the AYS program to improve patient outcomes.

### Supplementary Information

**Supplementary Material 1. **

**Supplementary Material 2. **

## Data Availability

All data generated or analyzed during this study are included in this published article and its supplementary information files.

## Abbreviations

AYS: Art Your Service
CAT: Creative Arts Therapies
EQ5D: EuroQoL-5 Dimension
eHEALS: EHealth Literacy Scale
LSNS: Lubben Social Network Scale
REB: Research and Ethics Board
SARS-COV-2: Severe acute respiratory syndrome coronavirus 2
SUS: System Usability Scale
