# Supplementary material for: Evaluation of an online arts-based platform to support the health and well-being of older adults during the COVID-19 pandemic: a cross-sectional survey
Source: BMC Public Health. 2024 May 4;24:1232. doi: 10.1186/s12889-024-18720-6 (PMC11069133; doi:10.1186/s12889-024-18720-6)
Supplement: Supplementary file 2 — Supplementary Material 2. [file 12889_2024_18720_MOESM2_ESM.docx]

**Appendix A**

**Art Your Service (AYS) Program Delivery**

**Supplemental Table 1:** AYS program delivery

| **Program Characteristics** | | **N (%)** | |
| --- | --- | --- | --- |
| **How you Heard About AYS (n=69)** | | | |
|  | Seniors’ Centre or Municipality | 31 (44.9) | |
|  | Friend or family | 22 (31.9) | |
|  | Other | 6 (8.7) | |
|  | Seniors’ Organization (e.g., CARP) | 5 (7.2) | |
|  | Newspaper | 4 (5.8) | |
|  | Website | 1 (1.4) | |
|  | Other:   - *Doctor”* - *“Community Care for Seniors, PEC”* - *“Study Group”* - *“Trial ad on LinkedIn”* - *“NYGH KeepWell Program”* - *“Local online news provider”* - *“I am thankful that I read about Art Your Service Program in the Toronto Star and happy to be a participant in the program.”* | | |
| **Duration of AYS Program Use (n=68)** | | | |
|  | 0-3 Months | 4 (5.9) | |
|  | 3–6 Months | 7 (10.3) | |
|  | 6-12 Months | 24 (35.3) | |
|  | 12 Months or More | 33 (48.5) | |
| **Frequency of AYS Program Use (n=68)** | | | |
|  | Less than once a month | 2 (2.9) | |
|  | Once a month | 4 (5.9) | |
|  | Twice a week | 23 (33.8) | |
|  | 3 or more times a week | 39 (57.4) | |
| **When is the AYS Program Used* (n = 71)** | | | |
|  | Mornings | 50 (46.30) | |
|  | Afternoons | 28 (25.93) | |
|  | Evenings | 6 (5.56) | |
|  | Weekdays | 17 (15.74) | |
|  | Weekends | 7 (6.48) | |
| **Live Participation vs. Recorded (n=68)** | | | |
|  | Live | 24 (35.3) | |
|  | Recorded | 6 (8.8) | |
|  | Live and recorded | 38 (55.9) | |
| **Goals, Needs and Reasons for Joining AYS Program* (n=67)** | | | |
|  | Improving my physical fitness | | 54 (80.6) |
|  | To learn something new | | 51 (76.1) |
|  | To help me get through difficult situations such as the COVID-19 pandemic | | 41 (61.2) |
|  | Improving my social and emotional wellbeing | | 35 (52.2) |
|  | Improving my mental wellbeing | | 35 (52.2) |
|  | Opportunities for interaction | | 30 (44.8) |
|  | Feeling isolated | | 28 (41.8) |
|  | Feeling bored | | 26 (38.8) |
|  | Feeling lonely | | 18 (26.9) |
|  | Desire for making social connections | | 16 (23.9) |
|  | Reputation of the program | | 15 (22.4) |
|  | Wanting company and/or making friends | | 14 (20.9) |
|  | Desire for emotional support | | 10 (14.9) |
| **Have your Goals been Satisfied (n=69)** | | | |
|  | Yes | | 60 (87.0) |
|  | Somewhat | | 7 (10.1) |
|  | No | | 1 (1.4) |
|  | I’m not sure | | 1(1.4) |

**Participants could select multiple responses.*

**Appendix B**

Perceptions of survey participants about the Art Your Service (AYS) Program delivery, impact on health and wellbeing and satisfaction

| **Characteristic** | **N** | **Item** | **Mean (SD)** | **Median** | **IQR*** |
| --- | --- | --- | --- | --- | --- |
| **Delivery** | 67 | I like that the sessions are delivered virtually (i.e., Zoom) | 4.61 (0.576) | 5 | 1 |
|  | 66 | The virtual classes are easy to access from the *Art Your Service* (AYS) platform | 4.64 (0.515) | 5 | 1 |
|  | 65 | The sessions have the right level of interaction and involvement | 4.48 (0.709) | 5 | 1 |
|  | 67 | The length of the virtual classes is just right | 4.52 (0.660) | 5 | 1 |
|  | 66 | The virtual classes are easy to understand | 4.61 (0.551) | 5 | 1 |
|  | 65 | I am comfortable participating in a virtual classroom environment | 4.49 (0.831) | 5 | 1 |
|  | 67 | The Program schedule is convenient for me | 4.40 (0.719) | 5 | 1 |
| **Impact on health and wellbeing** | 67 | I am learning new knowledge and skills | 4.18 (0.903) | 4 | 1 |
|  | 63 | I am applying what I have learned in my life | 4.10 (0.837) | 4 | 1 |
|  | 62 | The program helps my social wellbeing | 4.08 (0.893) | 4 | 1 |
|  | 64 | The program helps my mental wellbeing | 4.20 (0.858) | 4 | 1 |
|  | 67 | The program helps my physical wellbeing | 4.46 (0.725) | 5 | 1 |
|  | 63 | The program helps me to learn ways to take better care of my health | 4.10 (0.797) | 4 | 1 |
|  | 62 | I feel that my overall health has improved since I’ve joined the AYS Program | 4.08 (0.929) | 4 | 2 |
|  | 69 | I will continue to use the AYS Program | 4.58 (0.695) | 5 | 1 |
|  | 68 | I will recommend the AYS Program to my friends and family | 4.60 (0.672) | 5 | 1 |
| **Satisfaction** | 67 | Overall satisfaction with the Program | 4.72 (0.598) | 5 | 0 |
|  | 67 | Program organization (e.g., consistency; expected delivery) | 4.69 (0.556) | 5 | 1 |
|  | 67 | Administrative processes (e.g., subscription; emails of upcoming sessions) | 4.67 (0.660) | 5 | 0 |
|  | 64 | Instructors | 4.63 (0.630) | 5 | 1 |
|  | 67 | Quality of the Program content | 4.61 (0.602) | 5 | 1 |
|  | 67 | Program schedule | 4.55 (0.634) | 5 | 1 |
|  | 66 | Zoom video quality | 4.61 (0.579) | 5 | 1 |
|  | 64 | Pricing of the Program | 4.44 (0.774) | 5 | 1 |
|  | 66 | Variety of the virtual class topics | 4.39 (0.742) | 5 | 1 |
|  | 66 | Relevance of the virtual classes to what I am interested in | 4.35 (0.794) | 4.5 | 1 |
|  | 67 | Understandability of the content of the classes | 4.66 (0.509) | 5 | 1 |
|  | 67 | Pace of the session (i.e., not too fast, not too slow) | 4.46 (0.611) | 5 | 1 |
|  | 67 | Delivery of the class by instructors | 4.55 (0.658) | 5 | 1 |

*Interquartile range (IQR) of 0, 1 and 2 are considered high, good and poor consensus; respectively.

**Appendix C**

**AYS Program Content**

**Supplemental Table 2:** Summary of quantitative and qualitative survey responses regarding the AYS program content and instructors

| **Virtual Class Participation* (n=71)** | | **N (%)** |
| --- | --- | --- |
| Gentle Moves (Dance) | 44 (62.0) | |
| Joy of Movement | 44 (62.9) | |
| Pilates | 39 (54.9) | |
| Seated and Standing Fitness | 38 (53.5) | |
| Yoga and Meditation | 37 (52.1) | |
| Trivia Social | 33 (46.5) | |
| Cooking Classes | 32 (45.1) | |
| Creative Aging: Books and Ideas Series | 30 (42.2) | |
| Body Drumming | 25 (35.2) | |
| Mildly Spicy Moves/Zumba | 25 (35.2) | |
| Painting (Acrylic and Watercolor) | 25 (35.2) | |
| Drawing (Profiles, Cartoons) | 21 (29.6) | |
| Make a Move with Jess: Creative Movement | 20 (28.2) | |
| Zentangles | 20 (28.2) | |
| Music-based (e.g., Smile Theatre’s Tuney Tuesday, Ukulel) | 19 (26.8) | |
| Nutrition (e.g., Food and Mood) | 19 (26.8) | |
| Virtual Museum visits | 17 (23.9) | |
| Plants and Flowers | 17 (23.9) | |
| Socials (Antiques, Photos, Plant Social, Games, etc.) | 14 (19.7) | |
| Collages | 13 (18.3) | |
| Various Crafting | 11 (15.5) | |
| Sewing | 10 (14.1) | |
| Tech Support | 9 (12.7) | |
| Unexpected Acts of Kindness/Letter Campaigns | 6 (8.5) | |
| Poetry | 5 (7.0) | |
| Flash Memoirs | 1 (1.4) | |

| **Additional Suggested Topics** | **Board and Card Games:**   - Yahtzee - Bridge (card game) - More Trivia - Chess - More variety of games   **Financial Literacy:**   - Taxes - Banking tips for seniors   **Peer Connections:**   - Availability of a chat group   **Educational:**   - Metis - pronunciation of names, ways to address persons, learn about their traditions - Interesting professional lectures from doctors, astronauts etc. - Technology use for seniors   **Arts-Based:**   - How to play or play along guitar; recorder; harmonic - Drawing classes; learning how to draw - Art classes - Lettering for gift cards - Continued watercolor - i.e. specifically learning to watercolor parts - Art gallery and studio tours - Couch concerts   **Mindfulness:**   - Tips on overcoming boredom - Longer meditation sessions   **Physical Activity:**   - Osteofit - More cardio classes - Fitness class - Tai Chi - Seniors Zumba   **Nutrition:**   - Healthy nutrition - Cooking classes   **Hobbies/Interests:**   - Ancestry photography - Travel discussions - Animal-related |
| --- | --- |
| **AYS Program Sessions** | - *“I enjoy & look forward to the various programs especially fitness, cooking & social times. I'm not interested in art programs. I like that if you miss a program, you can always catch it from the library.”* - *“Downloading the recipes is helpful. I am not able to go to the grocery store and cook along. I do smile as I listen to others comment.”* |
| **AYS Program Session Instructors** | - *“The instructors are great.”* - *“I think the instructors do an excellent job of ensuring the content is relevant to all participants ... ie, using a chair versus standing. And more aggressive versus less aggressive. Well done!”* - *“It is excellent! They have the best staff and instructors! Kind, caring and very helpful. Everything is paced to the senior.”* - *“Social interaction with questions and general caring instructors who take the time to chat at the end of the activity.”* - *“The instructors are open to suggestions and happy to answer questions. No competition or criticism evident.”* - *“The instructors are all first class and emphasize the importance of movement. Questions can be asked at the end of every session.”* - *“Some questions are a bit difficult to answer, since we may have our favorite instructors. For me, one instructor has more time talking rather than moving than I would prefer. But other instructors manage this very, very well.”* |

**Participants could select multiple responses.*

**Appendix D**

**Advantages and Benefits of the AYS Program**

**Supplemental Table 5:** Quantitative survey responses about the advantages of participating in the AYS program

| **Advantage or benefit* (n = 65)** | **N (%)** |
| --- | --- |
| Doesn’t require travel | 60 (92.3) |
| Improves my physical health | 53 (81.5) |
| Flexible timing | 51 (78.5) |
| Ability to practice at own pace | 48 (73.8) |
| Ease of access | 47 (72.3) |
| Improves my cognitive health (thinking skills) | 41 (63.1) |
| Improves mental/psychological health (anxiety, depression) | 36 (55.3) |
| Improves my social health (loneliness, isolation, connectedness) | 34 (52.3) |
| Express oneself without worry | 25 (38.5) |
| Maintains privacy | 24 (36.9) |
| Provides respite from informal caregiving | 5 (1.2) |

**Participants could select multiple responses.*

**Supplemental Table 6:** Qualitative survey responses about the advantages of participating in the AYS program

| **Advantages and Benefits to Participating in the AYS Program** |
| --- |
| **Doesn’t Require Travel:**   - *“I hope the zoom continues after covid since I can’t get out.”* - *“Please keep these programs going, I don't drive now, so these programs give me something to keep me energized.”* - *“Excellent program...helps you stay connected even if you are homebound.”* - *“It has given me the opportunity to join many different types of programs without leaving my home. The old folks centre is good till December 31st. but I will continue after that, and it will be well worth my pension money… with so many programs to choose from.”* - *“Allows for social interaction for those living in rural areas.”*   **Self-Directed Access and Use:**   - *“Benefits are you can participate at your own pace or improvise if necessary for health issues. i.e. frozen shoulder, low iron - could only get back to doing part of a physical class for a time.”*   **Overall Benefits:**   - *“All the above really!”* - *“Just down right touches one’s soul.”* - *“It was a great help during COVID lockdowns and now I'm hooked!”* - *“I would love everyone to have access to this program - I think CAMH might find it helpful for some / many clients I intend to tell my doctor about this program. I just can’t say enough good things!”* - *“Sounds like it might be a great program for many people.”* - *“This is a wonderful program. I feel very happy to be part of it.”* - *“Doing a great service.”* - *“So glad I found AYS.”* - *“Excellent program! Keep up the good work!”* - *“Love the programs!!”* - *“Love it!”* - *“What Jen is doing is life changing. I’m so happy to be a part of the program.”* - *“It’s great!”* - *“Great program. I wish I had more time to participate more.”* - *“I believe that I would miss this programming if not present in my life. Hooray for ArtYourService!”* - *“I have spent so much time on this Program. I am so glad the Program was offered to me by my Municipality. Cannot express the huge difference the Program has made in my life. The Program is well organized, and every member is treated with respect and kindness.”*   **Program Continuity:**   - *“Keep this going please.”* - *“Hope it continues for many more years.”* - *“Really enjoy the art classes, hope they continue.”* - *“Excellent program! Keep up the good work!”* - *“Keep it up guys!”* - *“Please keep up the good work.”* - *“I hope Art Your Service Program will continue. Cannot imagine my life without it. Definitely need the exercise program. Enjoy learning new things i.e. ukulele, art. I do not enjoy going out for these programs and probably would not attend if had to go out for them.”* - *“I hope the zoom continues after covid since I can’t get out.”* - *“Please keep these programs going, I don't drive now, so these programs give me something to keep me energized.”*   **General Class Satisfaction:**   - “*There is quite a variety of programs to choose from that satisfy my interests.”* - *“Classes have been worthwhile.”* - *“Have enjoyed most of the programs I’ve seen. Do not take active part because of poor computer skills.”* - *“I am appreciative that I am able to have a group to go to and not have to worry about covid or lockdowns. It provides me with a health distraction. I feel motivated to do and try things. The author talks are very exciting. I already am reading one of the books.”* - *“Art Your Service has been a great way to continue to exercise and to learn new programs that I would have missed during the lockdown. They are so good that I really enjoy the variety of classes offered.”* - *“I enjoy & look forward to the various programs especially fitness, cooking & social times. I’m not interested in art programs. I like that if you miss a program, you can always catch it from the library.”* - *“It is user friendly, gives a great variety of classes.”* - *“Really enjoy the art classes, hope they continue.”* - *“The art and fitness were the only areas of interest to me.”* - *“The exercise classes are my priority. I often do not get around to 2 sessions a day, but do access the library later in the day for art, books, etc. (gardening, housekeeping, paperwork, etc. get in the way.)”* - *“I participate in the exercise classes. Have no comments concerning the afternoon events.”* - *“The art and fitness were the only areas of interest to me.”*   **Recommend to Others:**   - *“I have talked to many friends and family members about how wonderful I feel this program is. I have encouraged other seniors in my environment to participate, but many were not comfortable using Zoom. Because of the COVID-19 restrictions, they could not get any help setting up Zoom.”* - *“I have recommended the program to many family, friends and former students. Iam a yoga teacher unable to hold classes during covid, and I feel good being able refer people to such a good service. As a yoga teacher I feel I have been unable to meet their needs, that I have been one more disappointment during covid. Taking the zoom classes from Art Your Service has helped me get used to the technology and I am considering starting to teach a class online to a few former students. I am really missing the fulfillment that teaching gives me. I will of course continue to participate in AYS. It is such a comprehensive offering.”* |

**Appendix E**

**Challenges and Barriers to the AYS Program**

**Supplemental Table 3:** Quantitative survey responses about the challenges of the AYS program

| **Barrier or Challenge** | | **N (%)** |
| --- | --- | --- |
| **Challenges of AYS Program Use* (n=60)** | | |
|  | No difficulties | 37 (61.7) |
|  | Connectivity/network problems | 16 (26.7) |
|  | Sound quality | 10 (16.7) |
|  | Image quality | 4 (6.7) |
|  | Setting up/using software | 2 (3.3) |
| **Barriers or Challenges to Participating *(n=60)** | | |
|  | No barriers or challenges | 50 (83.3) |
|  | Not having social interaction with other participants | 6 (10) |
|  | Difficulties in accessing or using the technology | 4 (6.7) |
|  | Lack of quiet time to practice | 2 (3.3) |
|  | Not having one-to-one support and encouragement | 2 (3.3) |
|  | Lack of interaction with the instructor | 2 (3.3) |
|  | Lack of suitable space to practice | 1 (1.7) |
|  | Not having any social activities before/after class | 1 (1.7) |

**Participants could select multiple responses.*

**Supplemental Table 4:** Qualitative survey responses about the challenges of the AYS program

| **Technical Challenges in Accessing AYS** |
| --- |
| **Difficulties in Accessing AYS:**   - *“Missed getting the link occasionally but email Jen and receive right away. Thanks”*   **Difficulties with AYS Interface – Audio:**   - *“Sometimes difficult to hear; recording image is sometimes foggy.”* - *“I like music to be in the background so I can hear instructor better.”*   **Difficulties with AYS Interface – Video:**   - *“Sometimes difficult to hear; recording image is sometimes foggy.”* - *“The only thing I have noticed is when I watch a class from the archives, the video is only a partial screen. When I watch a live class, the complete screen is used.”* - *Difficulty reading chat messages due to vision limitations.”*   **User-related Difficulties:**   - *“Minor difficulties probably caused by local problems.”* - *“I experienced some network difficulty that my service provider has rectified.”* - *“A few connection issues”* - *“I am not always able to access the library. I receive a warning that the connection is not safe. I do not know how to solve this yet.”* - *“We have limited internet accessibility.”* - *“My old iPad had difficulty accessing the archived classes - but luckily my sister gave me a newer one!”* - *“Limited internet service”* - *“Wish I could call up on rerun the programs I missed, due to internet issues, forgetting the program was on until almost too late. And to check instructions”* - *“Hasn't had much of a chance due to limited internet allowance. Frequent service interruptions.”* |
| **Barriers or Challenges to Participating** |
| **Course Delivery Barriers:**   - *“The main reason I don't use this type of program is some members over lead the conversation during classes, discouraging others from participating.”* - *“Recently, I have been dissatisfied with the Friday morning exercise class, therefore I have stopped participating on those days. It is important that the instructors deliver classes in a senior friendly way.”*   **Scheduling Barriers:**   - *“Sometimes the timing is not convenient.”* - *“I just think the afternoon programs should start at 2.30pm.”* - *“More time options?”* - *“Have on weekends e.g. Saturday and maybe evenings.”* - *“Any of the movement sessions have been beneficial. The chair yoga is particularly helpful in maintaining healthy blood pressure. The interviews with authors have been very enjoyable. I would like to participate in some of the more social programs, but my caregiving duties don't allow for many real time sessions.”* - *“Great program. I wish I had more time to participate more.”* - *“The only reason I may not have participated in classes is when I have had other activities to replace them.”*   **Technology Use Barriers:**   - *“I think some people may have trouble with the technology at first, but once you've done it once or twice, it's easy!”* - *“I think that "fear of trying new things on a computer" is more of a barrier for seniors. I joined because a friend invited me. I have invited my friends but only one joined for a short while.”* - *“I have talked to many friends and family members about how wonderful I feel this program is. I have encouraged other seniors in my environment to participate, but many were not comfortable using Zoom. Because of the COVID-19 restrictions, they could not get any help setting up Zoom.”* - *“The questions about technology and ease of use are hard to answer - I am able to use it but - I do feel there may be seniors who need a little / or a lot, of guidance to get used to the zoom program. Such a personal situation —- But if they are using email - it is pretty easy. Vision issues might be a roadblock to seeing the directions on screen to access the classes —- I do know of a senior home where staff do the video set up and then chairbound clients participate.”* - *“Have enjoyed most of the programs I've seen. Do not take active part because of poor computer skills.”*   **Communication/Awareness Barrier:**   - *“I would like to have the programs record so I could see at more convenient time.”* - *“I just wondered if was live streaming all this time. Am I done yet???”* - *“Wish I could call up on rerun the programs I missed, due to internet issues, forgetting the program was on until almost too late. And to check instructions.”*   **Financial Barrier:**   - *“Due to my financial situation Jen has allowed me to participate fully at no cost… Greatly appreciated.”*   **Virtual Format Barriers:**   - *“There is a closeness that live interactions offer, that is different from what we are able to achieve on the internet.”* - *“I don't use my camera so others can see me (embarrassed), nor do I comment/ask questions technology and shyness issues questions.”* |
| **Technology Features:**   - *“I would like to be able to tap the class I'm interested in & have it "mark my calendar.”*   **Course/Class Content and Offerings:**   - *“No, except for maybe a couple of additional programs.”* - *“Purpose of art project not clear though process can be followed fairly easily. For example, explaining composition choices or colour mixing techniques so we have a better focus. Like to know why we are painting this picture...”* - *“I really can say it all otherwise in that I was interested in the art sessions, but I thought the subjects painted were rather odd.”*   **Technology Use Supports:**   - *“Open to everyone, and affordable for centres to subscribe to for their members. If it was paired with a Zoom information session and one on one workshops to help participants feel comfortable when joining if they need technological assistance.* |

**Appendix F**

Perceived Impact of the AYS Program

**Supplemental Table 7.** Summary of qualitative survey responses: impact of the AYS program on health, well-being and quality of life

| **Theme** | **Representative Respondent Quotes** |
| --- | --- |
| **Overall Health and Wellbeing** | - “*It has made me more aware of what I need to do in order to improve my health and wellbeing. I am definitely seeing very positive results from participating in this programme.”* - *“I have an interest in planning future activities again. Not just waiting for the days to pass. This gives me a feeling of wellbeing.”* |
| **Quality of Life** | - *“This is an outstanding service for seniors that improves the quality of life.”* |
| **Physical Wellness** | ***Physical Activity:***   - *“I am much more active thanks to Art Your Service.”* - *“It has kept me active, and brightened my spirits when I have the lost feeling after the loss of my husband after 55 years.”* - *“Getting me to move move move !!!!”* - *“Motivates me to move more and do extra healthy types of exercise.... e.g., lift weights.”* - *“Since my computer was down, I realized how much I missed the daily exercise and brain activities in the afternoon.”* - *“It’s making me more physically active & social during this Covid pandemic.”* - *“Helps me feel better about moving more and sitting less”* - *“I walk at least an hour each day, however, Art Your Service program offered me a variety of exercise each day which I like very much.”* - *“Learning “Jen Tangles” and the other art classes has taught me new avenues to explore, which I can do any time (and love). The fitness activities allow me to stay active.”* - *“The sessions were a godsend during the lockdowns. They provided a routine during the dreary winter months, and motivated me to do physical exercise.”* - *“Art Your Service has been a great way to continue to exercise and to learn new programs that I would have missed during the lockdown. They are so good that I really enjoy the variety of classes offered.”* - *“I continue to be active in front of a screen rather than being out of my home. I have settled that this is a new normal for me. The programming for the day lands in my inbox. I balance my day around the offerings for the day.”* - *“I love exercising virtually as I am not one that enjoys joining an exercise club.”* - *“Safe opportunity to engage in Physical activity during the pandemic.”* - *“Any of the movement sessions have been beneficial. The chair yoga is particularly helpful in maintaining healthy blood pressure. The interviews with authors have been very enjoyable. I would like to participate in some of the more social programs but my care giving duties don’t allow for many real time sessions.”*   ***Physical Fitness and strength:***   - *“I am stronger both physically and mentally!”* - *“They have made a great deal of change to my physical and therefore my mental wellbeing they have allowed to get the physical strength I require to continue to live on my own.”* - *“Participating in the Zoom sessions helped overcome the slight loneliness due to the lack of group activities in my retirement community. Physically, the sessions were a god send, as I am motivated by being part of a group.”* - *“I’ve been going through an ongoing health problem which they have not been able to figure out for the last 8 months and it’s been really necessary to keep up what I can manage in the classes to keep my muscles in shape!”* - *“I have learned how to easily get up from my bed.”* - *“My mobility has definitely increased immensely; I am able to care for myself so much better i.e., in tying my boots/shoes; reaching; not falling or being able to stop myself from a fall due to increased balance. Have so many more projects to work on ie art – from painting classes; playing ukulele.”* - *“I am much more flexible with no adverse aftereffects like sore muscles or restrained movement. I assisted a friend to move this week & I was surprisingly agile the next day, just a bit tired.”* - *“Major improvement in my pain management”* - *I have ‘met’ a variety of interesting and talented people and feel connected to the outside world. I have learned new and interesting skills that make me feel confident to try new things. I have empathy for others in different situations. I have maintained a level of fitness.”* - *“Better Physically.”* - *“I have a back problem and it has helped that under control.”*   ***Physical Health Self-Management:***   - *“Major improvement in my pain management”* - *“I have a back problem and it has helped that under control.”* - *“My mobility has definitely increased immensely; I am able to care for myself so much better i.e., in tying my boots/shoes; reaching; not falling or being able to stop myself from a fall due to increased balance. Have so many more projects to work on ie art - from painting classes; playing ukulele.”* |
| **Social Wellness** | ***Social Connectedness:***   - *“Excellent program...helps you stay connected even if you are homebound.”* - *“I enjoy being a part of this group.”*   ***Social Interaction***   - *“Allows for social interaction for those living in rural areas.”* - *“The Zoom sessions also helped me work through the recent loss of my husband by meeting new people while exercising.”* - *“It adds a structure to my life, that includes other people.”* - *Gave me a chance to be with different people, doing art.”* - *“Social interaction with questions and general caring instructors who take the time to chat at the end of the activity.”*   ***Social Activity***   - *“It has given me the opportunity to join many different types of programs without leaving my home. The old folks centre is good till December 31st. but I will continue after that, and it will be well worth my pension money… with so many programs to choose from.”*   ***Social Isolation***   - *“Lessen isolation.”*   ***Loneliness***   - *“Participating in the Zoom sessions helped overcome the slight loneliness due to the lack of group activities in my retirement community. Physically, the sessions were a godsend, as I am motivated by being part of a group.”* |
| **Emotional and Mental Health** | - *“It has kept me active and brightened my spirits when I have the lost feeling after the loss of my husband after 55 years.”* - *“The Zoom sessions also helped me work through the recent loss of my husband by meeting new people while exercising.”* - *“They have made a great deal of change to my physical and therefore my mental wellbeing they have allowed to get the physical strength I require to continue to live on my own.”* - *“The structure of having an exercise class each morning helps me get my day going. The library helps me be productive and relaxed when I need that.”* - *“I am stronger both physically and mentally!”* - *“This is a good program for me and one of the best things about it is....I feel it is a safe way to exercise....little risk of injury.”* |
| **Renewed Motivation in Life** | - “*Wanting to get back into my art.”* - *“Something to really look forward to in the monotony of the days. Tuning in at 10:30 assures me of quality exercise that I may or may not have put off.”* - *“I have an interest in planning future activities again. Not just waiting for the days to pass. This gives me a feeling of wellbeing.”* - *“A motivator to get up and look presentable daily.”* - *“Motivates me to move more and do extra healthy types of exercise.... e.g., lift weights.”* - *“I'm so pleased how these programs have kept me going on a daily basis since my move up north.”* - *“Since my computer was down, I realized how much I missed the daily exercise and brain activities in the afternoon.”* - *“Something to really look forward to in the monotony of the days. Tuning in at 10:30 assures me of quality exercise that I may or may not have put off.”* |

**Appendix G**

Perceived Impact of the AYS Program during the COVID-19 pandemic

**Supplemental Table 8.** Summary of qualitative survey responses: impact of the AYS program during the COVID-19 pandemic

| **Theme** | **Representative Respondent Quotes** |
| --- | --- |
| **A Sense of Routine, Structure and Purpose During Lockdowns** | *“It has been wonderful to get up in the morning and attend an easy-going exercise, then maybe at 2:00 pm take a relaxing painting class. It certainly filled in a gap; instead of watching tv all day and also learning new skills and hobbies, I will continue.”* |
|  | *“The daily programs provided structure and sense of purpose, especially during the lockdowns.”* |
|  | *“The sessions were a godsend during the lockdowns. They provided a routine during the dreary winter months, and motivated me to do physical exercise.”* |
|  | *“I have been able to attend classes while the Senior's Centre has been closed due to Covid. I like having structure, and attending classes provides that.”* |
|  | *“This program gave a structure to my week days - for months, like many at home, I gradually lost the need to get things done, to even get dressed each morning! Having a class each day, sometimes morning and afternoon really changed my attitude and inspired me to get involved. I tried art classes that I had wondered about for years - and really liked them. And I try art things even without the guidance of the teachers now. The boredom and sadness of the lockdown was so diminished the more classes I took. I felt busy and inspired, and purpose was back in my life.”* |
|  | *“At first AYS was a real substitute for my loss of participation in and outside the home activities -- I missed all the fun things I had been attending at the Seniors Centre and at church. I could join by zoom to such a variety of programs every day. Something to look forward to. Then I connected with others attending the classes, got to know them a bit, looked forward to seeing them. Shared a few laughs, etc.”* |
|  | *“I just really look forward to participating and learning. Otherwise, I would just be watching TV and talking to the dog, LOL.”* |
|  | *“Gives me something to look forward to every day during lockdowns”* |
|  | *“It has given me purpose and something to look forward to each week.”* |
|  | *Thanks for providing activities to make life meaningful.”* |
|  | *“I continue to be active in front of a screen rather than being out of my home. I have settled that this is a new normal for me. The programming for the day lands in my inbox. I balance my day around the offerings for the day.”* |
| **Accessibility and Convenience** | *“Great alternative since in-person classes are banned during covid.”* |
|  | *“I love exercising virtually as I am not one that enjoys joining an exercise club.”* |
|  | *“Safe opportunity to engage in Physical activity during the pandemic.”* |
|  | *“I have been able to attend classes while the Senior's Centre has been closed due to Covid. I like having structure, and attending classes provides that.”* |
|  | *“I send a smile your way for this program. Having virtual experiences for me is equal to in person experiences.”* |
|  | *“It was just wonderful to have these zoom programs available. My health has not gotten worse with the Covid-19 holding my own.”* |
|  | *“It's certainly helped to have scheduled interaction in these crazy times!”* |
|  | *“Really enjoy the classes and if unable to take the live class then accessing the library is an asset.”* |
|  | *“I travel a fair amount and can take my classes with me. Big advantage for me.”* |
|  | *“Love being able to access when I want :)”* |
|  | *“I personally feel that this is a great way to participate in classes as you set your own schedule and have access to the library when live class is missed.”* |
|  | *“I especially appreciated the fact that Jen was present at all activities, that she allowed participants into the Zoom sessions even when I was late. Access to recordings was very beneficial.”* |
| **Distraction from the COVID-19 Pandemic** | *“The daily programs provided something to do, a distraction from the ongoing news cycle [about COVID-19].”* |
|  | "I am appreciative that I am able to have a group to go to and not have to worry about covid or lockdowns. It provides me with a healthy distraction.” |
|  | *“Something to do since all my volunteer and exercise activities were cancelled.”* |
|  | *“Kept me focused from not always having Covid on my mind.”* |
|  | *“It helps me get through covid as well as it helps me temporarily escape from the senior’s residence where I live which have found very disappointing”* |
|  | *“Huge benefit during this pandemic.”* |
|  | *“Good diversion”* |
| **Something to Look Forward to** | *“Something to look forward to during the afternoons, as/an alternative to knitting in front of the TV.”* |
|  | *Gives me something to look forward to every day during lockdowns.”* |
|  | *It has given me something to look forward to from Monday to Friday. The variety of activities is excellent, and it is time well spent.”* |
|  | *It's given me something to look forward to, love learning new things.”* |
|  | *I just really look forward to participating and learning. Otherwise, I would just be watching TV and talking to the dog, LOL.”* |
|  | *“At first AYS was a real substitute for my loss of participation in and outside the home activities -- I missed all the fun things I had been attending at the Seniors Centre and at church. I could join by zoom to such a variety of programs every day. Something to look forward to. Then I connected with others attending the classes, got to know them a bit, looked forward to seeing them. Shared a few laughs, etc.”* |
| **Emotional Stability and Support** | *“Kept me sane and healthy. Helps me laugh and learn.”* |
|  | *“I have remained relatively sane during this pandemic, and I do believe that coming to this site has made a big difference for me.”* |
|  | *“It helps me get through covid as well as it helps me temporarily escape from the senior’s residence where I live which have found very disappointing.”* |
|  | *“I feel alive and wonderful. Thanks to your program.”* |
|  | *“It has kept me jovial and pleasant instead of old and crotchety....”* |
|  | *“It has helped me mentally, physically and emotionally respond to the covid pandemic in a positive manner and enriched my life beyond measure.”* |
|  | *“A bright spot in a dark time.”* |
|  | *“I feel happier.”* |
|  | *“Made things better.”* |
|  | *“I feel alive and wonderful. Thanks to your program.”* |
|  | *“It has kept me jovial and pleasant instead of old and crotchety....”* |
| **Filling a Void** | *“Something to do.”* |
|  | *“Fantastic……Thank you Jen for thinking of this idea. It has helped many, many people to pass the time when they have been shut in the house for the last 18 months.”* |
|  | *“At first AYS was a real substitute for my loss of participation in and outside the home activities -- I missed all the fun things I had been attending at the Seniors Centre and at church. I could join by zoom to such a variety of programs every day. Something to look forward to. Then I connected with others attending the classes, got to know them a bit, looked forward to seeing them. Shared a few laughs, etc.”* |
|  | *“Something to do since all my volunteer and exercise activities were cancelled.”* |
|  | *“It has been wonderful, just to get up in the morning and attend an easy-going exercise, then maybe at 2.00pm take a relaxing painting class. It certainly filled in a gap, instead of watching tv all day, and also learning new skills and hobbies I will continue. Also, Jen is wonderful if you have any computer related problems.”* |
|  | *“I just really look forward to participating and learning. Otherwise, I would just be watching TV and talking to the dog, LOL.”* |
| **Learning Something New** | *“I started doing things that I had not done before, and it was a way to stay involved while we were in lockdown. Still exercise with AYS every Monday morning. Love seeing my friends at that time too.”* |
|  | *“It's given me something to look forward to, love learning new things.”* |
|  | *“It keeps me busy and developed new interests.”* |
|  | *“I just really look forward to participating and learning. Otherwise, I would just be watching TV and talking to the dog, LOL.”* |
|  | *“Learning "Jen Tangles" and the other art classes has taught me new avenues to explore, which I can do any time (and love). The fitness activities allow me to stay active.”* |
|  | *“I started doing things that I had not done before, and it was a way to stay involved while we were in lockdown. Still exercise with AYS every Monday morning. Love seeing my friends at that time too.”* |
|  | *“This program has brought a lot of joy to my life...and the joy of movement and the stimulation of ongoing learning...I am learning to play the ukelele and I want to learn to paint. Priceless.”* |
|  | *“I have 'met' a variety of interesting and talent people and feel connected to the outside world. I have learned new and interesting skills that make me feel confident to try new things. I have empathy for others in different situations. I have maintained a level of fitness.”* |
|  | *“Art Your Service has been a great way to continue to exercise and to learn new programs that I would have missed during the lockdown. They are so good that I really enjoy the variety of classes offered.”* |
|  | *“I hope Art Your Service Program will continue. Cannot imagine my life without it. Definitely need the exercise program. Enjoy learning new things i.e. ukulele, art. I do not enjoy going out for these programs and probably would not attend if had to go out for them.”* |
|  | *“It's given me something to look forward to, love learning new things.”* |
|  | *“Learning "Jen Tangles" and the other art classes has taught me new avenues to explore, which I can do any time (and love). The fitness activities allow me to stay active.”* |
|  | *“This program has brought a lot of joy to my life...and the joy of movement and the stimulation of ongoing learning...I am learning to play the ukelele and I want to learn to paint. Priceless.”* |
| **Motivation to Maintain Health** | *“The sessions were a godsend during the lockdowns. They provided a routine during the dreary winter months and motivated me to do physical exercise.”* |
|  | *“I am appreciative that I am able to have a group to go to and not have to worry about covid or lockdowns. It provides me with a health distraction. I feel motivated to do and try things. The author talks are very exciting. I already am reading one of the books.”* |
|  | *“I found the morning exercises was a good excuse to get up in the morning.”* |
|  | *“Art Your Service has been a great way to continue to exercise and to learn new programs that I would have missed during the lockdown. They are so good that I really enjoy the variety of classes offered.”* |
|  | *“I continue to be active in front of a screen rather than being out of my home. I have settled that this is a new normal for me. The programming for the day lands in my inbox. I balance my day around the offerings for the day.”* |
|  | *“It has helped me mentally, physically and emotionally respond to the covid pandemic in a positive manner and enriched my life beyond measure.”* |
|  | *Please keep these programs going, I don't drive now, so these programs give me something to keep me energized.”* |
| **Opportunities for Social Interaction and Connectedness** | *“At first, AYS was a real substitute for my loss of participation in outside the home activities -- I missed all the fun things I had been attending at the Seniors Centre `` and at church. I could join by zoom such a variety of programs every day—something to look forward to. Then I connected with others attending the classes, got to know them a bit, looked for a sense of ward to seeing them. Shared a few laughs, etc.”* |
|  | *“I am grateful for the program. As a living alone senior, in seniors building, COVID-19 caused instant isolation.”* |
|  | *“The fact that I know there is somebody on the other side of the screen having the same need to connect with people and change ideas.”* |
|  | *“ [The AYS Program] helped me connect with people in a fun way”* |
|  | *“Before Covid I would have at least 6 lunches out amongst others, go on bus trips & cruises so AYS fills a gap in my social life.”* |
|  | *Before Covid I would have at least 6 lunches out amongst others, go on bus trips & cruises so AYS fills a gap in my social life.”* |
|  | *“Something to do since all my volunteer and exercise activities were cancelled.”* |
|  | *“Amazing to have this program to connect with others.”* |
|  | *“Something enjoyable and important happened every day. One gets to know people who have become virtual friends. And, of course, there is Jen Tindall who makes it all possible.”* |
|  | *“Helped me connect with people in a fun way.”* |
|  | *“I have met a variety of interesting and talent people and feel connected to the outside world. I have learned new and interesting skills that make me feel confident to try new things. I have empathy for others in different situations. I have maintained a level of fitness.”* |
|  | *“The program was quite handy to have during COVID. I have a strong social network.”* |
